# An ethnobotanical survey of wild edible plants of Paphos and Larnaca countryside of Cyprus

**DOI:** 10.1186/1746-4269-2-34

**Published:** 2006-09-04

**Authors:** Athena Della, Demetra Paraskeva-Hadjichambi, Andreas Ch Hadjichambis

**Affiliations:** 1 Agricultural Research Institute, P.O. Box 22016, 1516 Nicosia, Cyprus

## Abstract

An ethnobotanical survey of wild edible plants of Cyprus was carried out in two sites. Paphos vine zone and Larnaca mixed farming zone. These are among the areas in Cyprus whose inhabitants subsisted primarily on pastoralism and agriculture and therefore still preserve the traditional knowledge on wild edible plants.

The information was collected for three-year period, in the framework of the EU-funded RUBIA Project. Four hundred and thirteen interviews have been administered to 89 informants of various ages and background categories in 29 villages of Paphos site, and 8 in Larnaca site. A total of 78 species were recorded. Ethnographic data related to vernacular names, traditional tools and recipes have also been recorded. A comparison of the data collected from the two sites is undertaken. During this ethnobotanical research it was verified that wild edibles play an important role in Cyprus in rural people, however, it was realized that the transmission of folk uses of plants decreased in the last generations. The research of ethnobotany should be extended to other areas of Cyprus in order not only to preserve the traditional knowledge related to plants but to make it available to future generations as well.

## Background

Even though covering only 9251 square kilometres, Cyprus is a country diverse in geography, climate, flora and fauna and rich in history and culture. Cyprus is the third largest island in the Mediterranean with a climate of wet, changeable winter and hot dry summers, separated by short spring and autumn seasons of rapidly changing weather. The vegetation of Cyprus is formed by typical Mediterranean types: the coniferous forest, the maquis, the garigue and the batha vegetation, whilst more localized communities occur around salt marshes, sand dunes, stone walls and mountain streams [1-4].

In Cyprus, about 2000 taxa were recorded as native or naturalized. From the native taxa, 143 were recorded as endemics [5-8]. References to the Cyprus flora and in particular to plants of economic importance go back as far as Homer. Cyprus' plants are mentioned in the works of ancient authors such as Theophrast, Dioscourides and Pliny. Among Cyprus natural vegetation, a number of aromatic, medicinal and other useful plants are being exploited in their wild form [9].

The Cyprus diverse topography has permitted the survival of traditional knowledge related to vegetable resources used by locals as food. Even though, the consumption of plants gathered from the wild represented an important part of human nutrition in Cyprus, however, there are few ethnobotanical studies focused on wild edibles [10-13].

The present research was performed in the framework of the EU-funded RUBIA Ethnobotanical Project (Contract Number ICA3–2002–10023, 2003–2006). The perspectives of this research project were to record ethnobotanical knowledge related to traditional plant uses of wild and neglected cultivated plants for food, medicine, textiles, dyeing, handicrafts, and basketry, as well as to identify and evaluate the socio-economic and anthropological context in which these plants have been gathered and processed.

As a part of this broad study, wild food plants have been recorded in Cyprus and therefore the aim of this paper, is to present and analyze the wild food data gathered in the study areas of Cyprus during the years 2003–2005.

## Methodology

### Location and study area

**Figure 1:**
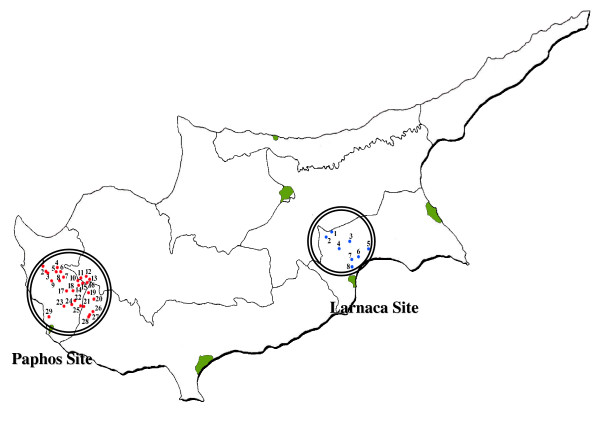
Map of Cyprus with the two study sites.

Within Cyprus, two areas of study have been selected for this research project, according to the Agro-economic zones of Cyprus [14]. The decision was made in order to fulfil the criteria set by the EU-RUBIA Consortium for rural areas administratively, geographically and ecologically homogeneous with similar socio-economic context (Figure 1).

In both sites man transformed the natural landscape, in order to create opportunities for agriculture and stock raising. The floral diversity of the territories (especially in Paphos area) and the different ways in which their inhabitants have exploited the natural resources available have engendered a rich popular knowledge of the use of plants. Not ethnobotanical studies have been carried out in these regions until now.

Site one belongs to the 4^th ^phytogeographical zone of Cyprus, which has mostly cultivated or heavily grazed land in the North and numerous barren, eroded chalk or limestone hills in the South [3]. Is a part of Larnaca mixed farming zone and is an area of 155 km^2 ^consisting of 8 relatively big villages: Athienou, Avdhellero, Kellia, Livadhia, Petrophani, Pyla, Troulli, Voroklini, with in total 9545 inhabitants all of whom are autochthonous Greek-Cypriots, Greek speaking with Cypriot dialect. Cereals are the main crops planted, however the low irrigation of the area and the limited profitability of cereals compelled the farmers to concentrate mostly to livestock production.

Site two belongs to the 1^st ^phytogeographical zone of Cyprus, which is an area heterogeneous topographically, geologically and floristically, with much natural vegetation. It is mostly hilly, with deep narrow gorges, limestone or sandstone and with interesting areas of serpentine [3]. Site two is a part of the Paphos vine zone and is an area of 375 km^2 ^comprising 29 small villages: Axylou, Amargeti, Agios Demetrianos, Dhrinia, Dhrousia, Eledhio, Inia, Kallepia, Kannaviou, Kathikas, Kato Akourdhalia, Kelokedara, Pano Arodes, Panayia, Choulou, Kritou Marottou, Lemba, Letymbou, Melemiou, Miliou, Pano Akourdhalia, Phiti, Polemi, Psathi, Stroumbi, Theletra, Tsada, Yiolou, Pitagrou, with 9540 inhabitants all of whom are autochthonous Greek-Cypriots, Greek speaking with Cypriot dialect and Paphian idiom. Even though the region extends over a large area with many villages, there is a small number of inhabitants in each village and it is considered the less densely populated region of the country. The major crop planted is the grape vine followed by cereals [14]. Part of the western site of this territory has been suggested for inclusion in the Akamas Natura 2000 site.

These two sites are among the few areas in Cyprus whose inhabitants subsisted primarily on pastoralism and agriculture and therefore the older people of these areas still preserve the traditional knowledge on wild edible plants. The intensity of farming and the unavailability of off-farm job opportunities were closely related to the population engaged in agriculture. Today, most of the young people of both sites work in Paphos or Larnaca towns, leaving the agricultural and pastoral activities to be carried out by the middle-aged and older generations.

The interest of the present study was focused on wild food botanicals in the two sites. Attempts have been made to correlate and compare the plants recorded between the two sites as well as with other research work carried out in Cyprus and abroad.

A further aim of this research was to develop an ethnobotanical framework which could be the basis for further studies.

## Methods

The present research was performed in the framework of the EU-funded RUBIA Ethnobotanical Project. The aim of this research project was the recording of ethnographical field data in order to develop a model for the re-evaluation of tools and technologies related to traditional uses of wild and neglected cultivated plants for food, medicine, textiles, dyeing, handicrafts, and basketry, as well as to identify and evaluate the socio-economic and anthropological context in which these plants have been gathered and processed. Eight study areas from the following countries were participated: Algeria, Cyprus, Egypt, Greece, Holland, Italy, Morocco and Spain.

The field methodological framework chosen for this research was that used in ethnobiology [15-17]. Field research was conducted by collecting ethnobotanical information during structured and semi-structured interviews with knowledgeable people native in each site territory. For each plant recorded one questionnaire was filled. Even though a structured questionnaire had to be filled direct questions were avoided. The basic information needed was taken during the conversation. Whenever possible the conversation was recorded on cassettes.

No special selection criteria were used in the choice of the informants because one of the aims of this work was to assess the breadth of popular heritage in the field of wild edible plants, knowledge which is widespread among locals. However, most of the interviewees were more than 60 years old, and belong mainly to families which have a strong connection with traditional agricultural activities.

Plant data and their related information were entered into a data base. The data acquired for each plant comprise the common local name, its uses, the part of the plant used and its preparation and administration processes. The way plants were collected, preserved, stored, prepared and used and the most relevant processes were photographed and video recorded.

Most of the mentioned plants were recognised by the villagers *in-situ *during short field walks and collected for scientific identification. Nomenclature followed mainly the *Flora of Cyprus *[3,4] and in some cases the *Flora Europaea *[18]. Herbarium specimens of most of the taxa cited were prepared and deposited in the National herbarium of Cyprus at the Agricultural Research Institute, Nicosia. Seed samples were also collected in the appropriate season for the most representative wild plants and deposited in the Cyprus National Genebank, at the Agricultural Research Institute.

## Results/discussion

Four hundred and two interviews have been administered to 89 informants, of which 38 (43%) were women and 51 (57%) were men. Informants were between the ages of 48–82, with the average age of 66.

A total of 78 plants have been recorded. All these species are native and are gathered from the wild whilst 11 of them are cultivated as well (*Ceratonia siliqua*, *Eruca sativa*, *Mentha spicata*, *Origanum dubium*, *Rosmarinus officinalis*, *Thymus capitatus*, *Laurus nobilis*, *Ficus carica*, *Myrtus communis*, *Portulaca oleracea*, *Crataegus azarolus*). Comparing the plants recorded in the two sites it can be seen that 40 plants are common in both sites, 5 of the edible plants are used exclusively in Larnaca site and 33 plants are used exclusively in Paphos site. Within the two sites the dependency of rural people on agriculture was much greater in the Paphos vine zone than in Larnaca site. According to studies of 1983 [14] in Paphos site 71% of rural people were gainfully employed in agriculture and 29% in other occupation whilst in Larnaca site 43% of people employed in agriculture and 57% in other occupation. The closer relation of the indigenous people with their land probably resulted to the higher degree of usage of the natural plant resources in Paphos site. Additionally, many villages in Paphos site are near or within the Akamas Nature Reserve, a big area with many natural habitats and rich vegetation and therefore many of the wild edibles are gathered from the undisturbed shrublands of the area. Furthermore, the middle-aged generation of the Paphos vine zone, even though working in the town, they have relation with the countryside, still gaining profits from their grapes, and therefore still preserve some of the TK of their parents.

The survey of wild edible plants of Paphos and Larnaca countryside is the first study in Cyprus which has followed ethnobotanical methodology, recording not only a species list but ways of gathering, storage, preservation, preparation processes, common and traditional recipes and therefore the comparison of our data with previous studies is not possible. However, an attempt was made in order to compare only the species list of wild edibles recorded in our two study areas with the list of edible wild plants of the Cyprus Flora published in 2000 which enlisted 57 edible species from all around the island [13]. From the comparison was revealed that 47 plants were recorded in both species lists, 29 wild edibles were reported for the first time in our ethnobotanical study and 10 species were recorded only in Savvides' list and not in ours.

All the plants recorded are presented in Table 1 with the indication of scientific name, vernacular name, family, plant part used, type of preparation, site recorded, number of records and herbarium specimen number.

### Most used plants

The recorded plants belong to 31 different families. Asteraceae was with difference the most frequently encountered botanical family with 20 taxa, whilst Apiaceae and Brassicaceae follow with seven taxa, Lamiaceae with six and Boraginaceae is represented by four taxa. The other 26 families have less representation between one to three taxa each. Most of them are big families with many representatives in the Mediterranean region, some of which are very common plants. The data of this study confirm that people tend to use preferably the plants that are easily available to them excluding of course, those that are toxic or noxious. As was affirmed by other publications as well [19-22], the more common a plant (family or species) is in an area, the greater is the probability of its popular use. As for the most known and used species 13 of them were cited 10 times or more. The food utilization of *Centaurea hyalolepi*s, has been reported by 18 informants, followed by *Silene vulgaris *(17 citations), *Capparis spinosa *(16 citations), *Thymus capitatus *(16 citations), *Asparagus acutifolius *(15 citations), *Malva parviflora *(14 citations), *Scolymus hispanicus *(13 citations), *Eryngium creticum *(12 citations), *Foeniculum vulgare *(11 citations), *Onopordum cyprium*, *Carlina involucrata *ssp. *cyprica *and *Portulaca oleracea *with 10 citations each. A high number of plants (49 out of 78) have been recorded by at least three independent informants, so that they follow the reliability criterion of Le Grand and Wondergem [23] and would be particularly interesting in view of further studies [22].

**Figure 2:**
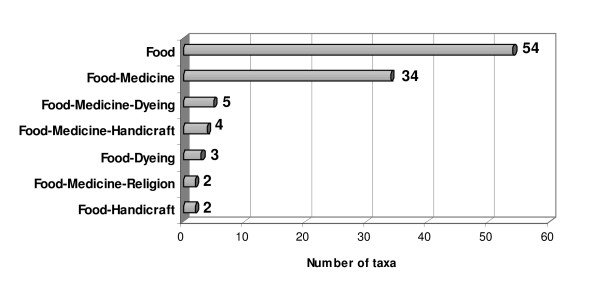
Number of plants used for food and other uses.

At this point it should be noted that 40 of the edible plants recorded are used exclusively for food. Some other plants have two or more uses and they appear in different categories as well. As can be seen in figure 2, 37 (30+4+3) plants have been recorded to be used for food as well as for medicine.

This overlap indicates the close relationship between health and food. A good example to this is *Origanum dubium*. The origan, locally called "rigani", is one of the most commonly edible plants used and many traditional recipes were recorded for its use as a condiment such as in recipes of roasted meat, as a scent in kebab, and is added as a scent in a traditional recipe, called "tsamarella" which is made from salted goat meat. It is also considered one of the most commonly used medicinal with about six different recipes, against flu, cold, as antipyretic, anti-stress, for stomach-ache and good digestion. These plants (*Origanum dubium*, *Thymus capitatus*, *Laurus nobilis*, among others) are often used in folk medicine as digestive, so it may be that their presence in these often heavy dishes is not only a culinary but medicinal, to increase the digestibility of the cooked food [19]. Overlapping between foods and medicines is quite well known in traditional societies [24-26] and represents an often neglected field in ethnopharmaceutical research.

### Plant supply/availability throughout a year

Most of the plants are collected in wild populations nearby the places where the informants live. Occasionally there is a small-scale cultivation in their home gardens (*Origanum dubium*, *Myrtus communis*, *Crataegus azarolus*). Some plants which were very much appreciated and frequently consumed in the past are now considered as weeds and even though have been mentioned they are only rarely eaten; in the territories studied this is the case of *Sinapis alba *and *Sinapis arvensis*. *Sinapis *spp. are still eaten in other areas of Cyprus [13].

Among all the edibles, four endemic species of Cyprus were recorded. The presence of endemic species illustrates the fact that the informants have a deep knowledge of their environment, since the three of them are not very abundant and can be found only in certain areas. For example, the endemic subspecies *Carlina involucrata *ssp. *cyprica *and *Centaurea calcitrapa *ssp. *angusticeps *are used only from the inhabitants of specific villages in Paphos area whilst the endemic *Origanum majorana *var. *tenuifolium*, which is used like common oregano, can be found only in a shrubland area of the Akamas National Park. The endemic species *Onopordum cyprium*, is used both in Paphos and Larnaca site and is a very common plant (Figures 6 and 7).

One of the favourite edibles of the recent past, *Gundelia tournefortii*, known by locals in Paphos area as "silifa", is under threat since it has become rare and it can not easily be found. This plant has been included in the Red data book of Cyprus Flora as Endangered because its populations have been eliminated [27].

Most wild species are gathered from waste and uncultivated land (48%) or from shrubland (17%) and by the roadside (12%). Eight percent (8%) of wild edibles are grown within or around the cultivations and therefore can be collected from the cultivated land of grape vines in Paphos and Cereals in Larnaca,

In the local Cyprus cuisine, greens and wild plants in general, have an important role. According to this study during winter, it is possible to use 49 wild plants, and this number can increase to 56 during spring. The number then decreases and in May many edible greens have bloomed and the leaves have become tough, leaving only about 16 still edible. During summer some fruits of wild trees are edible.

**Figure 3:**
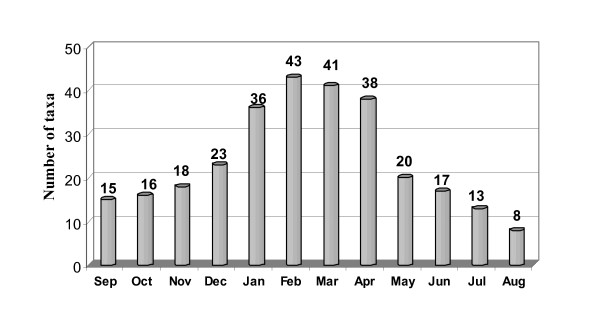
Availability of wild edible plants throughout a year.

From these plants only 15 can be purchased throughout a year from local markets and stores (*Capparis spinosa, Ceratonia siliqua*, *Cynara cornigera, Eruca sativa, Mentha spicata, Origanum dubium, Rosmarinus officinalis, Pistacia lentiscus, Silene vulgaris, Thymus capitatus, Laurus nobilis, Ficus carica, Myrtus communis, Portulaca oleracea, Crataegus azarolus*). These plants are partly collected from the wild and partly coming from small scale cultivation. Some of them are used as a condiment, some others are consumed as greens in salads or they are used for the preparation of cooked recipes. The other 66 taxa people should gather only from the wild by themselves (Figure 3).

As regards the tools used for gathering, 44% of the plants are gathered simply by hand while 37 % are gathered by a knife. Other tools such as a big knife (9%), a traditional big curved knife called "skylloua" (7%) and scissors (3%) are also used.

#### 4.3 Plant parts

**Figure 4:**
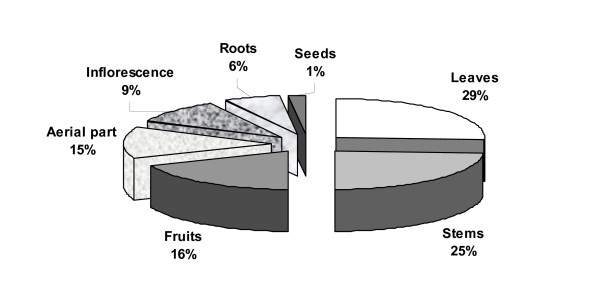
Plant parts most widely used.

Within the edible plants, leaves (29%) and stems (25%) are the plant parts most widely used. Fruits and aerial part follow with 16% and 15% respectively (Figure 4).

Among the recorded plants thistles are very popular as wild edibles of Cyprus. The young stems of 16 wild plants are used. Eight of them are used in both sites (*Centaurea hyalolepis*, *Scolymus hispanicus*, *Scolymus maculatus*, *Onopordum cyprium*, *Eryngium creticum*, *Cynara scolymus*, *Echinops spinosissimus*, *Notobasis syriaca*, while seven of them are used exclusively in Paphos site (*Centaurea calcitrapa *ssp. *angusticeps*, *Silybum marianum*, *Cynara cardunculus*, *Carlina involucrata *ssp. *cyprica*, *Carduus argentatus *ssp. *acicularis*, *Gundelia turnefortii*, *Onopordum bracteatum*) and one of them is used exclusively in Larnaca site (*Cynara cornigera*). These plants can be gathered from January to March, and their young stems, cleaned of spines, are used in most cases boiled with legumes or fried.

### Models of consumption

**Figure 5:**
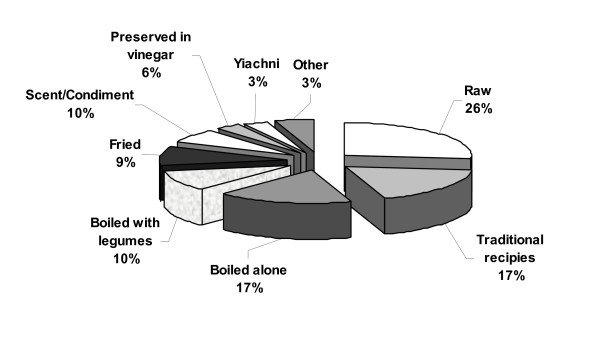
Models of edible plants consumption.

The edible plants are consumed in many different ways. Some of them need only the washing of the part of the plant to be eaten, and some others imply a more or less complex preparation process (Figure 5).

#### Raw

Many plants (26%) with edible leaves, roots or fruits are eaten raw. Many of them are used in salads. This is the case of *Portulaca oleracea*, *Ammi majus*, *Apium nodiflorum*, *Taraxacum cyprium*, *Capsella bursa-pastoris*, *Foeniculum vulgare*, *Mentha pulegium *which are usually dressed with oil, and vinegar or lemon. Some others like *Sinapis alba*, *Sinapis arvensis*, *Taraxacum hellenicum*, *Cichorium intybus*, *Nasturtium officinale*, *Sonchus oleraceus*, *Allium neapolitanum *are eaten fresh with olives, onions and bread. On the other hand, many edible fruits are directly consumed as desserts, in fresh form (*Pyrus syriaca*, *Crataegus azarolus*, *Crataegus monogyna*, *Ziziphus lotus*). The existence of *Limonium sinuatum *in this group is remarkable, because it is the first time that this plant is cited as a food plant in Cyprus [13] even though has been listed as an edible for the Mediterranean in Bodrum area of Turkey [29].

**Figure 6:**
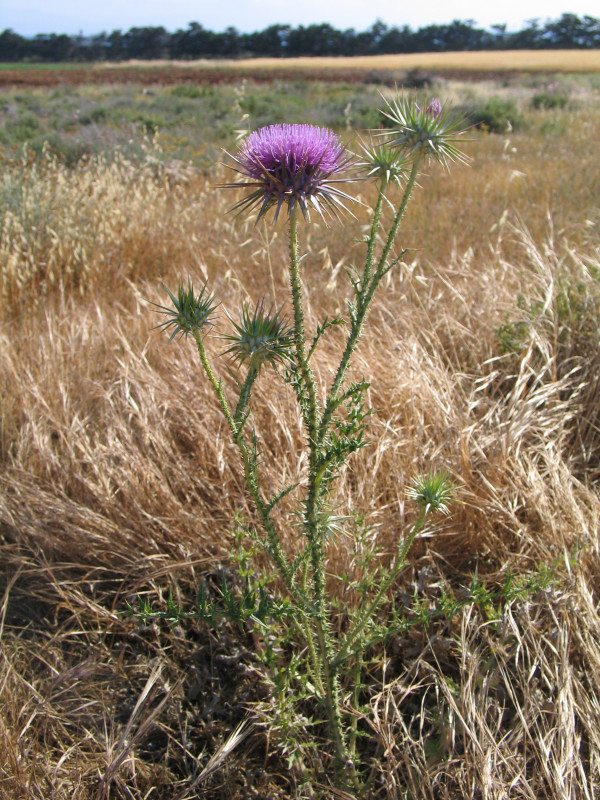
*Onopordum cyprium *Eig.

**Figure 7:**
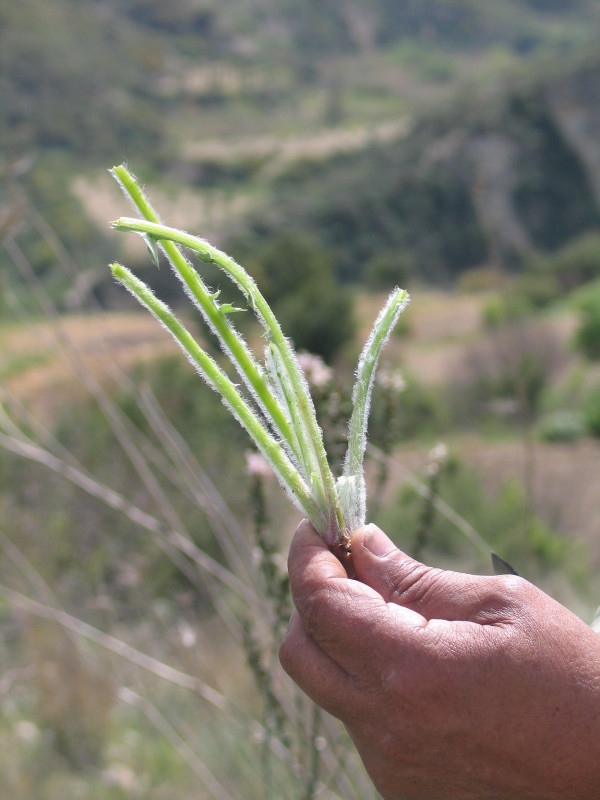
Collection and clean-up of thorns of the endemic edible plant *Onopordum cyprium *Eig.

#### Cooked plants

A number of wild plants (59%) are eaten cooked. Most of them, 27 %, are eaten boiled, 17% are eaten boiled alone and 10% are eaten boiled with legumes, especially with broad beans. In both cases they are garnished with olive-oil and lemon. The most popular plants used as boiled are: *Centaurea hyalolepis*, *Scolymus hispanicus*, *Carlina involucrata *ssp. *cyprica, Malva parviflora*. However, some more elaborated preparations were recorded. Some plants are consumed fried (9%) and especially in an omelette. The young shoots of *Asparagus acutifolius*, *Asparagus stipularis *and the young leaves of *Silene vulgaris*, which are the most typical examples in both sites studied, are cut, fried and mixed with the eggs to make the omelette. *Asparagus acutifolius *is prepared in the same way in some parts of Italy [28] the Iberian Peninsula [19] and in Bodrum area of Turkey [29].

A number of wild edibles (17%) is used in traditional recipes. It is worth mentioning that very popular among traditional recipes in Cyprus are home made pies, called in general "pittes". Eleven plants are used for making traditional pies. First, dough is made from flour, water and salt and then it is used for making small pies. Some times the pies are filled with the boiled or fried leaves (*Beta vulgaris *ssp. *maritima*, *Papaver rhoeas*, *Silene vulgaris*, *Rumex pulcher*) along with rice or "pourgouri" (like couscous) and spices. Some other plants are used as a scent in the pie (*Foeniculum vulgare*, *Mentha spicata*). In other cases fruits raw or preserved are used as the main filling of the pie (*Pistacia lentiscus*, *Pistacia terebinthus*, *Ficus carica*). Pies are cooked in the oven. Wild plants can also be a basis for a soup, the most famous of which is the so-called "molochosoupa" (malva soup), made with *Malva parviflora*. Some plants are often cooked in a traditional recipe called "yiachni" meaning, fried with onions and then tomato juice is added. Finally, plants are sparsely a condiment or the complement of meat stews as occurs with *Cynara cardunculus*, *Cynara cornigera *and *Gundelia tournefortii*.

#### Preserved plants

A number of plants are gathered and preserved to be stored and consumed all year round. Many plants which are used as a scent are dried and stored in plastic bags, plastic bottles or glass vessels and therefore used all year round. Nine plants (10%) are used to condiment stews, soups, pies or other dishes and traditional recipes. The most popular aromatic plants are *Origanum dubium*, *Mentha spicata*, *Rosmarinus officinalis*, *Laurus nobilis*, *Thymus capitatus*, *Origanum majorana *var. *tenuifolium, Foeniculum vulgare*. These plants add a distinct flavour and aroma to pies as well as to meat stews. *Rosmarinus officinalis *is used in a traditional fish recipe called savoro.

Some other plants such as *Capparis spinosa*, *Crithmum maritimum*, *Eryngium creticum*, *Eryngium glomeratum *and *Muscari comosum*, are preserved in vinegar and eaten like appetizer with several kind of food. Fruits of several wild trees are used for the preparation of jams and marmalades such as *Pyrus syriaca*, *Crataegus azarolus *and *Crataegus monogyna*.

Many tools used in processing were recorded. The five tools more often recorded are: "Madratzi", a traditional wooden long tool for opening pies, "Chti and Chtocheri", a traditional copper pot used for pounding, "Chartzi", a traditional copper pot used for boiling, "Satzi", a metal hot plate used for cooking pies, "Koumna", a traditional jar used for storage and "Gastra", an earthen vessel used for storage.

## Conclusion

This study carried out in two sites of Cyprus showed that the habit of using edible wild plants is still alive, but is "ageing". The consumption of wild plants is done as an addition or a complement to a diet of cultivated food plants. During this ethnobotanical research it was verified that even though wild edibles has been playing an important role in Cyprus since ancient times, it was realized that the transmission of folk uses of plants decreased in the last generations and surely in urban areas the knowledge is very much delimited. Almost all the interviewees, were past retirement age, and agreed that today far fewer wild plants are consumed than in previous decades. The people of the younger generation we met during the field survey declared that "*it is much easier and less time and effort consuming to buying greens, fruits or spices from the markets, no matter if they are cultivated or even imported, instead of running to the fields. Since even though, going to the wild it is not easy to recognise the edible plants and in case can identify some of them they are not familiar with the way plants should be processed"*. It is obvious that the younger generation has all but lost the TK necessary to identify, gather and process these species, while many middle-aged informants perceive the consumption of non-cultivated vegetables in a negative way, often as a symbol of poverty of the past.

The data of this study agree with those from other authors [30,31,19], and confirm that non cultivated edible plants deserve to be more thoroughly surveyed from an ethnobotanical and economic-botanical viewpoint, as a basis for agricultural, nutritional and other studies which may lead to the use of some new or renewed food plants. When studying wild food plants from this point of view, we must give recognition to the contribution of rural societies to the diversification of the sources of human nutrition and work for the reappraisal of folk knowledge on plants and their uses [32,33,19].

Our study, as well as other studies in a Circum-Mediterranean level [34,35], demonstrated that there is an urgent need for documentation of TK related to the intangible cultural heritage concerning traditional plant uses, and that such a heritage is much more complex that we may think. The ethnobotanical research should be extended to other areas of Cyprus in order not only to preserve the traditional knowledge related to plants but to make it available to future generations as well, showing the way for authenticity, simplicity and revival of that which is genuine.

All authors read and approved the final manuscript.

## Supplementary Material

Additional File 1 Wild edible plants of the Paphos and Larnaca countryside of Cyprus. The species list of wild edible plants consumed in Paphos and Larnaca countryside of Cyprus including the plant parts used, type of preparation, site recorded, number of records and herbarium specimen number. Click here for file
