# Supplementary material for: An ethnobotanical survey of wild edible plants of Paphos and Larnaca countryside of Cyprus
Source: J Ethnobiol Ethnomed. 2006 Sep 4;2:34. doi: 10.1186/1746-4269-2-34 (PMC1599709; doi:10.1186/1746-4269-2-34)
Supplement: Additional File 1 — Wild edible plants of the Paphos and Larnaca countryside of Cyprus. The species list of wild edible plants consumed in Paphos and Larnaca countryside of Cyprus including the plant parts used, type of preparation, site recorded, number of records and herbarium specimen number. [file 1746-4269-2-34-S1.pdf]

Additional file/Table 1. Wild edible Plants of Paphos and Larnaca countryside of Cyprus (TR: traditional recipe)

Family	*Scientific name*	*Vernacular name (Greek, Cyprus dialect)*	*Plant part used*	*Type of use*	*Site recorded*	*Specimen No*	*No of mentions*
Alliaceae	*Allium ampeloprasum* L.	Agrioskordo, Agriopraso	Bulb	1. Raw with olives<br>2. Boiled with legumes	Paphos	ARI 5739	2
	Allium neapolitanum Cyr.	Skortalia, Agrioskordo	Bulb	1. Raw with olives<br>2. Boiled with legumes	Paphos	ARI 5743	2
Amaranthaceae	*Amaranthus graecizans* L.	Glindos, Vlito	Young stems, Leaves	1. Boiled alone<br>2. Boiled with legumes	Larnaca / Paphos	ARI 5899	4
Anacardiaceae	*Pistacia atlantica* Desf.	Tremithos, Tremithkia	Fruits	1. Raw<br>2.The traditional Paphian chewing gum<br>3. Oil	Paphos		2
	Pistacia lentiscus L.	Skinos, Schinies, Skinaki	Fruits	1. Pies (TR)<br>2.Sausages (TR)<br>3.Skinolado (oil)	Larnaca / Paphos	ARI 5625<br>ARI 5693	6
	Pistacia terebinthus L.	Tremithia	Fruits, Young stems	1.Tremythenes, pies (TR)<br>2.Fried tremythkia (TR)<br>3.Tremytholado (TR)<br>4. Edges of fresh stems are eaten raw or cooked	Larnaca / Paphos	ARI 5627<br>ARI 5654<br>ARI 5695	7
Apiaceae	*Ammi majus* L.	Arkoseleno	Young stems	1. Boiled<br>2. Boiled with legumes<br>3. Fried<br>4. Raw<br>5. Scent<br>6. Salad	Larnaca / Paphos	ARI 5862	3
	Apium nodiflorum (L.) Lag.	Arkoseleno	Young stems	1. Boiled<br>2. Boiled with legumes<br>3. Fried<br>4. Raw<br>5. Scent<br>6. Salad	Larnaca / Paphos	ARI 5896	3
	Bunium ferulaceum Sib.&Sm.	Stavrokaria	Tuber	1. Raw	Paphos	ARI 5894	3

	Crithmum maritimum L.	Kirtamo	Young stems, Leaves	1. In vinegar<br>2. Raw	Paphos	ARI 5567	2
	Eryngium creticum Lam.	Pagkalos	Young stems, Leaves	1. Raw<br>2. Salad<br>3. Boiled<br>4. In vinegar	Larnaca / Paphos	ARI 5608, 5616,5668, 5717	12
	Eryngium glomeratum Lam.	Pagkalos	Young stems, Leaves	1. Raw<br>2. Salad<br>3. Boiled<br>4. In vinegar	Larnaca / Paphos	ARI 5895	6
	Foeniculum vulgare Mill.	Marathos	Young stems, Leaves	1. Spice<br>2. In eliotes<br>3. As a scent<br>4. With potatoes<br>5. Salad<br>6. In vinegar<br>7. Yiachni<br>8. In kolokotes (TR)<br>9. In spanakopita (TR)<br>10. Raw with olives	Larnaca / Paphos	ARI 5561<br>ARI 5605<br>ARI 5687<br>ARI 5745	11
Asparagaceae	*Asparagus acutifolius* L.	Agrelia	Young stems	1. Boiled<br>2. Fried<br>3. Barbeque	Larnaca / Paphos	ARI 5789	15
	Asparagus stipularis Forssk.	Agrelia, Sparangia	Young stems	1. Boiled<br>2. Fried<br>3. Barbeque	Larnaca / Paphos	ARI 5884	8
Asteraceae	*Calendula arvensis* L.	Tsakrotiroues, Ftyrokaenes	Aerial part	1. Boiled<br>2. Meatballs (TR)	Larnaca / Paphos	ARI 5814	7
	Carduus argentatus L. ssp. *acicularis* (Bert.) Meikle	Kouroumbes, Maurocouroumbes	Aerial part	1. Boiled with legumes<br>2. Boiled alone	Paphos	ARI 5862	2
	Carlina involucrata Poir. ssp. *cyprica* Meusel et Kastner	Plotarka	Leaves, Stems	1. Raw<br>2. Boiled<br>3. Boiled with legumes	Paphos	ARI 5890	10
	Centaurea calcitrapa L. ssp. *angusticeps* (Lindberg f.) Meikle	Atrachoua, Trisatzia, Atrachia, Agratzia	Young stems	1. Boiled with legumes<br>2. Boiled alone	Paphos	ARI 5629	3
	Centaurea hyalolepis Boiss.	Atrachouna, Trisatzia, Atrachia	Young stems	1. Boiled with legumes<br>2. Boiled alone	Larnaca / Paphos	ARI 5773	18
	Chrysanthemum segetum L.	Similia	Aerial part	1. Raw<br>2. Boiled	Larnaca / Paphos	ARI 5863	4
	Cichorium intybus L.	Agrioradikia	Aerial part, Young stems,	1. Raw<br>2. Boiled alone	Larnaca / Paphos	ARI 5666	4

			Leaves	3. Boiled with legumes<br>4. In vinegar			
	Cynara cardunculus L.	Kafkaromana, Kafkaroua, Agrioagkinara, Arkotzynara	Leaves, Succulent receptacle	1. Boiled<br>2. Raw<br>3. Yiachni<br>4. Fried	Paphos	ARI 5624<br>ARI 5558	5
	Cynara cornigera Lindley	Kafkaromana, Kafkaroua, Kinara, Chosti	Young stems, Succulent receptacle	1. Raw<br>2. Yiachni<br>3. Boiled<br>4. Boiled with legumes<br>5. Fried	Larnaca	ARI 5864	3
	Echinops spinosissimus Turra	Saratzinos	Young stems	1. Raw<br>2. Boiled<br>3. Fried<br>4. With meat	Larnaca / Paphos	ARI 5782<br>ARI 5799	5
	Gundelia tournefortii L.	Silifa	Young stems, Inflorescence	1. Yiachni<br>2. Boiled with legumes	Paphos	ARI 5563	2
	Notobasis syriaca (L.), Cass	Nerokavlos, Gaourokavlos, Patsalokavlos	Leaves, Young stems	1. Raw<br>2. Boiled<br>3. Boiled with legumes	Larnaca / Paphos	ARI 5673<br>ARI 5726	3
	Onopordum bracteatum Boiss. & Heldr.	Asprangatho	Young stems, Succulent receptacle	1. Raw<br>2. Boiled<br>3. Boiled with legumes	Paphos	ARI 5866	2
	Onopordum cyprium Eig.	Gaedouragkatho	Young stems, Succulent receptacle	1. Raw<br>2. Boiled<br>3. Boiled with legumes	Larnaca / Paphos	ARI 5579,<br>ARI 5592<br>5684, 5552	10
	Scolymus hispanicus L.	Galatouna, Alatouna, Christagkatho	Young stems, Leaves	1. Raw<br>2. Boiled<br>3. Fried<br>4. Fried with eggs<br>5. Yiachni	Larnaca / Paphos	ARI 5727<br>ARI 5689<br>ARI 5719	13
	Scolymus maculatus L.	Plotarka, Galaktites, Aspragkatho, Atrachounes	Young stems, Leaves	1.Raw<br>2.Boiled<br>3. Fried	Larnaca / Paphos	ARI 5574<br>ARI 5600<br>ARI 5617	10
	Silybum marianum (L.) Gaertner	Nerokavlos, Gaourokavlos, Patsalokavlos	Leaves, Young stems	1. Raw<br>2. Boiled<br>3. Boiled with legumes	Paphos	ARI 5867	2
	Sonchus oleraceus L.	Tsiofos, Sonchos, Tsionchos	Aerial part	1. Raw in salads	Larnaca / Paphos	ARI 5808<br>ARI 5821	6
	Taraxacum cyprius Lindberg fil.	Agrioradikia, Agrioraditzia	Leaves	1. Raw	Paphos	ARI 5869	2

	Taraxacum hellenicum Dahlst.	Agrioradikia, Agrioraditsia	Leaves	1. Raw<br>2. In salads<br>3. Boiled	Larnaca / Paphos	ARI 5868	3
	Tragopogon sinuatus Ave.-Lall.	Kalakatsouna	Stems	1. Boiled alone<br>2. Boiled with legumes	Paphos	ARI 5673	2
Boraginaceae	*Anchusa italica* Rerz.	Oglossos, Otziokos	Aerial part	1. Boiled alone<br>2. Boiled with legumes<br>3. Fried<br>4. Raw	Paphos	ARI 5872	3
	Anchusa strigosa Labill.	Oglossos, Voudoglosso	Aerial part	1. Boiled alone<br>2. Boiled with legumes<br>3. Fried<br>4. Raw	Paphos	ARI 5725<br>ARI 5748	6
	Borago officinalis L.	Boratsino	Aerial part, Leaves	1. Boiled 2. In vinegar	Paphos	ARI 5873	2
	Echium angustifolium Mill.	Meloua, Melissotrofiko	Inflorescence	1. Children used to take the nectar from the flowers.	Paphos	ARI 5871	2
Brassicaceae	*Brassica nigra* (L.) Koch	Sinapi	Seeds	1. As a scent in Mougra (TR), in mustard, in pickles	Paphos	ARI 5664	2
	Capsella bursa-pastoris (L.) Medik.	Poudgi tou voskou, Agriogardamouda	Aerial part	1. Raw in salad	Larnaca / Paphos	ARI 5816	3
	Eruca sativa Mill.	Roca	Leaves, young stems	1. Raw in salad	Larnaca / Paphos	ARI 5816	3
	Erucaria hispanica (L.) Druce	Lapsana, Kotsinolapsana	Aerial part	1. Raw	Larnaca	ARI 5877	2
	Nasturtium officinale R. Br	Kardama, Kartamilla	Young stems, Leaves	1. Raw in salads	Paphos	ARI 5564<br>ARI 5567<br>ARI 5618	3
	Sinapis alba L.	Lapsana	Aerial part	1. Boiled alone<br>2. Boiled with legumes<br>3. Yiachni<br>4. Raw	Larnaca / Paphos	ARI 5672, 5681, 5742	10
	Sinapis arvensis L.	Lapsana	Aerial part	1. Boiled alone<br>2. Boiled with legumes<br>3. Yiachni<br>4. Raw	Paphos	ARI 5878	9
Capparidaceae	*Capparis spinosa* L.	Kappari	Young stems, Floral bud, Fruits	1. In vinegar (pickled)	Larnaca / Paphos	ARI5550<br>5583, 5591<br>5598, 5614	16

Caryophyllaceae	*Silene vulgaris* (Moench) Garcke	Stroufouthkia, Tsakridia, Strouthi	Leaves, Young stems	1. Fried with eggs.<br>2. Fried with beans.<br>3. Boiled with legumes<br>4. Pourekouthkia: (TR)	Larnaca / Paphos	ARI 5667<br>ARI 5694	17
Chenopodiaceae	*Beta vulgaris* L. ssp. *maritima* (L.) Arcang.	Agrioteftlo, agriolachano	Leaves	1. Boiled with legumes<br>2. Boiled alone<br>3. Pourekia me Lachanouthkia (TR)	Paphos	ARI 5875	2
Convolvulaceae	*Convolvulus althaeoides* L.	Afka tis Perdikas, Kampanoula, Perikoklai	Inflorescence	1. Raw	Larnaca	ARI 5714	2
Fabaceae	*Ceratonia siliqua* L.	Teratsia, Charoupia	Fruits	1.Teratsomelo (TR)<br>2.Toumachia (TR)<br>3.Koulourouthkia (TR)<br>4.Garnish of pilaf (TR)<br>5. Carob honey<br>6. Pasteli, Pastellaki (TR)	Larnaca / Paphos	ARI 5569	6
Fagaceae	*Quercus infectoria* Olivier ssp veneris (A. Kerner) Meikle	Valanidia	Fruits	1. Roasted	Paphos	ARI 5665	2
Hyacinthaceae	*Muscari comosum* (L.) Mill.	Agriohyacinthos	Bulb	1. Boiled<br>2. In vinegar	Paphos	ARI 5885	2
Lamiaceae	*Mentha pulegium* L.	Brizolo	Leaves	1. Raw in salads	Paphos	ARI 5628	2
	Mentha spicata L.	Dyosmos	Leaves	1. Ravioles (TR)<br>2. Scent in pourekia (TR)	Larnaca / Paphos	ARI 5880	6
	Origanum dubium Boiss.	Rigani	Inflorescence, Leaves	1. Scent in Tsamarella, Kebab (TR)	Paphos	ARI 5655	8
	Origanum majorana L. var. *tenuifolium* Weston	Sapsissia,	Inflorescence, Leaves	1. Scent in recipes	Paphos	ARI 5603	7
	Rosmarinus officinalis L.	Lasmari, Dentrolivano	Leaves, Young stems	1. Scent in Savoro (TR)<br>2. Scent in Zalatina (TR)	Larnaca / Paphos	ARI 5575<br>ARI 5581	5
	Thymus capitatus (L.) Hoffsgg. et Link	Thymari, Throumbi	Inflorescence, Aerial part	1. Scent in Haloumia (TR) and other recipes	Larnaca / Paphos	ARI 5806	16
Lauraceae	*Laurus nobilis* L.	Daphni	Leaves	1. Scent in Stiphado (TR) and other recipes	Larnaca / Paphos	ARI 5882	3
Malvaceae	*Malva parviflora* L.	Molocha, Molochoua	Leaves, Stems, Aerial part	1. Boiled alone<br>2. Fried<br>3. Soup (TR)<br>4. Molochosoupa (TR)	Larnaca / Paphos	ARI 5547	14

	Malva sylvestris L.	Molocha	Young leaves	1. Boiled alone<br>2. Fried<br>3. Soup (TR)	Larnaca / Paphos	ARI 5590	3
Moraceae	*Ficus carica* L.	Sykia	Fruits	1. Raw<br>2. Sikopites (TR)<br>3. Dry figs<br>4. Zivania (TR)	Larnaca / Paphos	ARI 5898	8
Myrtaceae	*Myrtus communis* L.	Myrtia, Mersinia	Fruits	1. Raw	Larnaca / Paphos		5
Oxalidaceae	*Oxalis pes-caprae* L.	Xiniatos, Xinoudi	Young stems, Leaves	1. Raw	Larnaca / Paphos	ARI 5888	3
Papaveraceae	*Papaver rhoeas* L.	Paparouna, Peteinos, Kotsinopetinos	Young stems	1. Fried<br>2. Boiled<br>3. Pourekia peteinou (TR)	Larnaca / Paphos	ARI 5682, ARI 5713	3
Plumbaginaceae	*Limonium sinuatum* (L.) Miller	Thalasokrampi	Young stems, Leaves	1. Raw with olives	Paphos	ARI 5568	2
Polygonaceae	*Rumex pulcher* L.	Xinidia, Oxinaeos, Xiniato, Glossa tis petheras	Aerial part	1. Xinopoureka (TR)<br>2. Boiled with broad beans	Paphos	ARI 5675<br>ARI 5736<br>ARI 5744	4
Portulacaceae	*Portulaca oleracea* L.	Glystirida	Leaves	1. Raw in salads	Larnaca / Paphos	ARI 5889	9
Rhamnaceae	*Ziziphus lotus* (L.) Lam	Konnara, Konnarka	Fruits	1. Raw	Larnaca / Paphos	ARI 5582<br>ARI 5551	4
Rosaceae	*Crataegus azarolus* L.	Mosfilia	Fruits	1. Raw<br>2. Marmelade	Larnaca / Paphos	ARI 5573, 5650, 5713	4
	Crataegus monogyna Jacq.	Kotsinomosfilia	Fruits	1. Raw<br>2. Marmelade	Paphos	ARI 5890	2
	Pyrus syriaca Boiss.	Arkappis	Fruits	1. Raw	Paphos		2
Ruscaceae	*Smilax aspera* L	Arkovatos	Young stems	1. Boiled<br>2. Fried alone or with eggs	Paphos	ARI 5886	3
Solanaceae	*Solanum nigrum* L.	Fyto Ai Giorkou	Fruits	1. Raw	Larnaca	ARI 5817	2
	Solanum villosum Mill	Pomiloroua	Fruits	1. Raw	Paphos	ARI 5892	2
Ulmaceae	*Celtis australis* L.	Konnarka	Fruits	1. Raw	Paphos	ARI 5893	2
